# Peanut 9-*cis*-epoxycarotenoid Dioxygenase Enhances Salt and Drought Stress Tolerance by Regulating ROS Homeostasis

**DOI:** 10.3390/plants14121741

**Published:** 2025-06-06

**Authors:** Wenlin Wang, Mo Zhou, Shaohui Xu, Zhong Huang, Xiaobei Li, Cai Zhou, Siming Wang, Kaiyuan Zhang, Lixian Qiao, Yanyan Tang

**Affiliations:** College of Agronomy, Qingdao Agricultural University, Qingdao 266109, China; wenlinwang2023@163.com (W.W.); 15104199779@163.com (M.Z.); xushaohui225@163.com (S.X.); 19845918185@163.com (Z.H.); 15053859262@163.com (X.L.); zc2839203830@163.com (C.Z.); wangsimingkkk@163.com (S.W.); 18329982521@163.com (K.Z.)

**Keywords:** peanut, 9-*cis*-epoxycarotenoid dioxygenase, *AhNCED4*, ROS scavenging system, salt and drought tolerance

## Abstract

Peanut (*Arachis hypogaea* L.), a vital oilseed and cash crop, faces yield limitations due to abiotic stresses. The 9-*cis*-epoxycarotenoid dioxygenase (NCED) enzyme, a key enzyme in abscisic acid (ABA) biosynthesis regulating plant development and stress responses, remains mechanistically uncharacterized in peanut abiotic stress tolerance. In this study, we isolated a novel gene, *AhNCED4*, from the salt-tolerant mutant M24. The expression of *AhNCED4* was strongly induced by NaCl, PEG6000, and ABA in peanut huayu20. Overexpression of *AhNCED4* enhanced salt and drought tolerance in *Arabidopsis*. Transgenic overexpression of *AhNCED4* improved salt and stress resistance through upregulated ROS-scavenging genes superoxide dismutase (SOD), peroxidase (POD), and catalase (CAT) with elevated enzymatic activities while reducing malondialdehyde (MDA), superoxide anion (O^2−^), and hydrogen peroxide (H_2_O_2_) accumulation compared to wild-type plants. Further research showed that the chlorophyll fluorescence parameters of transgenic lines were significantly increased, while light damage was significantly reduced. These findings establish *AhNCED4* as a critical regulator of stress adaptation and an excellent candidate gene for resistance breeding in peanut.

## 1. Introduction

Peanut (*Arachis hypogaea* L.), an important oil and cash crop, is extensively cultivated in more than 100 countries and is highly valued by consumers globally [1,2,3]. As well as serving as an excellent source of edible oils, peanut also act as a high-quality ingredient in food industry applications. In 2023, global peanut cultivation spanned approximately 30.92 million hectares, producing around 54.27 million metric tons, with China alone contributing over 19.23 million metric tons (without being shelled) [4]. Drought and salinity represent critical abiotic stresses that induce osmotic/oxidative stress, disrupt cellular ion/redox homeostasis, and increasingly threaten global food sustainability [5,6,7,8,9,10,11]. To cope with these stresses, plants employ defense mechanisms involving gene expression regulation, with enzymes and proteins serving as key mediators of abiotic stress tolerance through reactive oxygen species (ROS) scavenging, ion balance maintenance, and cell membrane integrity preservation [12,13].

The plant enzyme 9-*cis*-epoxycarotenoid dioxygenase (NCED), a pivotal component in abscisic acid (ABA) biosynthesis, catalyzes the stereospecific cleavage of 9-*cis*-epoxycarotenoids to generate xanthoxin—the essential ABA precursor [14,15,16,17]. NCED mediates the upregulation of endogenous ABA biosynthesis to enhance plant stress resistance under adverse environmental conditions [18,19,20,21,22]. NCEDs are members of a multigene family detected in many plant species. *NCED* homologs have been identified and studied in many plant species [23,24,25,26]. The first *NCED* gene was identified through the characterization of specific oxidative cleavage in the maize *vp14* mutant [27]. In *Brassica napus*, the expression of *BnNCED3* enhances stress tolerance by enhancing endogenous ABA biosynthesis and the production of NO and ROS in transgenic *Arabidopsis* [28]. In cotton (*Gossypium hirsutum* L.), the expression of *GhNCED5*, *GhNCED6*, and *GhNCED13* was similar to changes in ABA content, confirming their functional roles in ABA synthesis [25]. In rice, overexpression of *OsNCED3* results in reduced relative water loss, delayed seed germination, and greater drought tolerance relative to that of wild-type plants [29]. In wheat, heterologous expression of *TaNCED1* in tobacco significantly improved its drought tolerance, and it exhibited a higher germination rate and higher relative water content when compared with WT plants [30].

Research on the regulation of ABA biosynthesis has focused on stress responses in vegetative-stage plants across various species, as both environmental stimuli and developmental cues critically influence abscisic acid production in plants. The equilibrium between ABA biosynthesis and catabolism tightly regulates endogenous ABA concentrations. Notably, 9-cis-epoxycarotenoid dioxygenase (NCED) is recognized as a pivotal regulatory enzyme in ABA production, as its transcriptional activity strongly correlates with cellular ABA levels, and its overexpression directly induces ABA overaccumulation [31]. In *Arabidopsis* plants, overexpressing *AtNCED3* displays enhanced water stress tolerance accompanied by increased ABA levels [32]. After drought treatment, overexpression of *OsNCED3* results in increased accumulation of ABA [29]. Most importantly, the *nced3* mutant shows defective ABA biosynthesis [15]. While the functions of ABA metabolic genes are well-characterized in model plant systems, their identification and functional validation in peanuts are still important.

Peanut, a globally crucial oilseed crop, displays a certain level of tolerance to abiotic stress, though productivity remains significantly constrained by drought and salinity. *NCED* genes are established regulators of plant stress responses; however, their functional roles in peanut remain uncharacterized. The NCED gene family is rate-limiting in abscisic acid (ABA) biosynthesis, a central regulator of plant stress responses. However, the evolutionary conservation and functional divergence of NCED homologs in peanut remain poorly characterized, particularly under abiotic stresses. Analysis of the peanut transcriptome dataset comparing ‘Huayu20’ and the salt-tolerant mutant M24 [33] revealed that NCED4 exhibited the most significant expression changes, suggesting its potential involvement in salt stress tolerance. This study aimed to identify the *NCED* gene associated with salt and drought tolerance in peanut and validate its role in ROS scavenging through heterologous expression. This study elucidates the molecular characteristic of *AhNCED4* under salt and drought stress conditions in peanut.

## 2. Results

### 2.1. The Expression Analysis of the AhNCED4 Gene in Peanut

**Figure 1:**
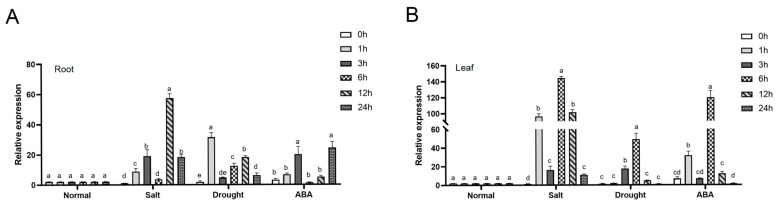
Expression analysis of *AhNCED4* gene in Huayu20. The expression levels of *AhNCED4* were assessed in roots (**A**) and leaves (**B**) before and after treatment with H_2_O, 30% PEG6000, 300 mM NaCl, and 100 μM ABA. Data are presented as mean ± SE (*n* = 3). Different letters indicate significant differences between treatment groups at the same time point.

In peanut, a novel *NCED* gene was isolated from a salt-tolerant peanut mutant M24 through reanalysis of transcriptome data (9-*cis*-epoxycarotenoid dioxygenase gene 4). Subsequent expression analysis in ‘Huayu20’ under NaCl, 30% PEG6000, and ABA treatments showed distinct induction patterns. In roots, *AhNCED4* expression peaked at 6 h, 1 h, and 24 h after NaCl, PEG, and ABA treatments, respectively (Figure 1A). Leaves displayed more pronounced responses, with expression levels reaching 67.28-fold, 47.37-fold, and 5.14-fold increases over controls following 6 h NaCl, 30% PEG6000, and 100 μM ABA treatments (Figure 1B). These results demonstrate that *AhNCED4* might play an important role in the response to salt, drought, and ABA stress.

### 2.2. Cloning and Sequence Analysis of AhNCED4

**Figure 2:**
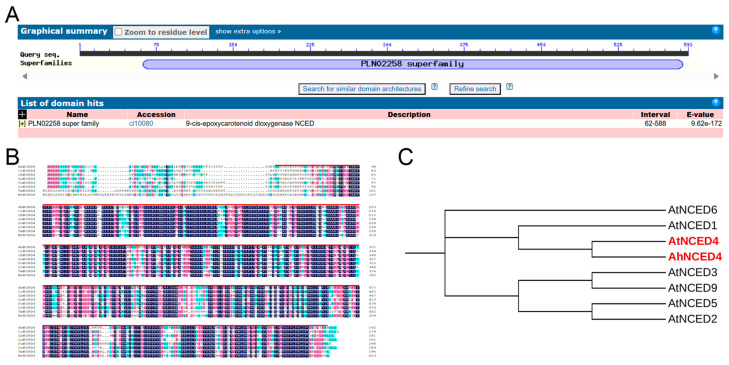
Sequence alignment and phylogenetic analysis of AhNCED4 proteins. (**A**) In the NCED protein sequence 62–588aa, there was a conserved domain called the PLN02258 superfamily. (**B**) Sequence alignment: Homologous NCEDs from seven different plant species, including *Cajanus cajan*, *Gastrolobium bilobum*, *Lupinus angustifolius*, *Populus alba*, *Stylosanthes scabra*, *Tripterygium wilfordii,* and *Morella rubra*. The conserved domain is marked by red lines. (**C**) Alignment of the amino acid sequences of AhNCED4 and its 7 orthologs.

*AhNCED4*, which showed strong induction under salt and drought stresses, was cloned from the salt-tolerant peanut mutant M24. The DNA sequence of this gene was 2970 bp, and it contained two exons and one intron. The full-length cDNA of *AhNCED4* was 1779 bp, and it featured a complete open reading frame encoding a 593 amino acid polypeptide with a predicted molecular weight of 148.46 kDa and a pI of 4.88 (Table S1). The encoding protein of AhNCED4 contained a conserved EXT signature domain, as demonstrated through multiple sequence alignment with NCED4 homologs from other species (Figure 2A,B). In addition, based on phylogenetic analysis, this novel protein shared the highest identity with AtNCED4 and was named AhNCED4 (Figure 2C).

### 2.3. Overexpression of AhNCED4 Enhanced Salt and Drought Tolerance in Arabidopsis

**Figure 3:**
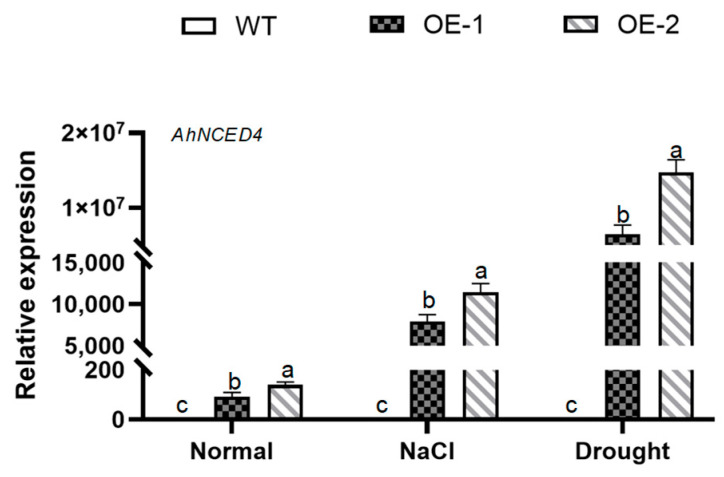
Expression analysis of the *AhNCED4* gene was performed in WT and overexpressing lines. The expression levels of *AhNCED4* were assessed in leaves before and after treatment with H_2_O, 30% PEG6000, and 300 mM NaCl. Data are presented as mean ± SE (*n* = 3). Different letters indicate significant differences between the same treatment groups.

To investigate the function of *AhNCED4* in plants, the expressed vector of this gene was transformed into WT *Arabidopsis*. A total of twelve independent homozygous *Arabidopsis* lines of *AhNCED4* overexpressing were obtained. T_3_ seeds from two confirmed transgenic lines (designated OE-1 and OE-2) were selected for subsequent analysis under salt and drought stress conditions. Under normal conditions, the expression level of *AhNCED4* was significantly elevated in these two overexpressing lines. Moreover, under both salt and drought stress conditions, its expression was even more markedly higher than that in the wild-type plants (Figure 3).

**Figure 4:**
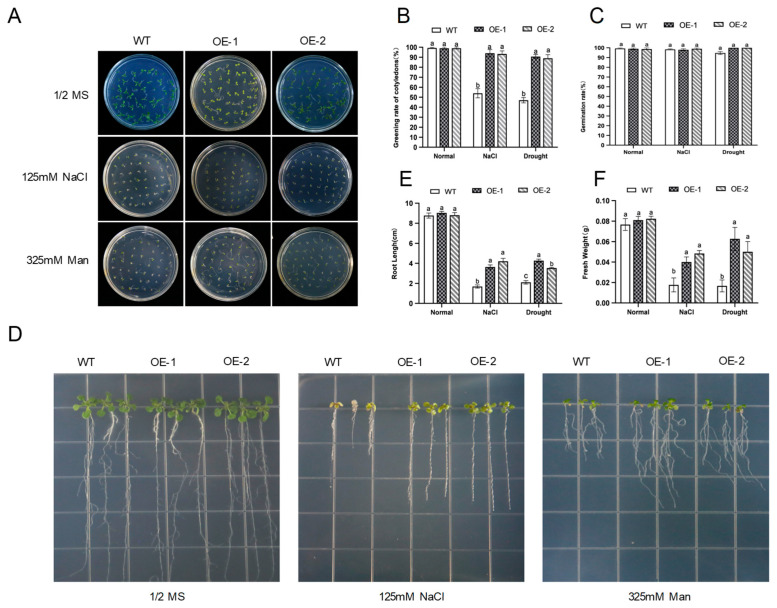
The seed germination phenotype, seedling surface morphology, and physiological trait variations in WT and transgenic *Arabidopsis* under normal, salt stress, and drought stress conditions. (**A**) The seed germination phenotype of WT and transgenic *Arabidopsis* under 125 mM NaCl and 325 mM mannitol treatments. (**B**) The phenotypes of transgenic lines and WT seedling plants after one week of growth on 1/2 MS medium supplemented with 125 mM NaCl and 325 mM mannitol. (**C**,**D**) The cotyledon greening rate (**C**) and germination rates (**D**) were calculated between WT and transgenic *Arabidopsis* under salt and drought treatments, respectively. (**E**,**F**) The primary root length (**E**) and fresh weight (**F**) of transgenic lines and WT plants were measured, respectively. Different letters indicate significant differences between the same treatment groups.

To determine the germination rate of *AhNCED4* under salt and drought stresses, 1/2 MS medium containing 125 mM NaCl or 325 mM mannitol was employed for salt or drought stresses for seven days, respectively. Under control conditions, all the seeds from the WT and transgenic lines could be fully germinated after sowing, with no significant difference in germination rate (Figure 4A–C). However, the germination and cotyledon greening rates were significantly higher in the transgenic lines than in the WT plants under the stress treatments (Figure 4A–C). Based on the results of the root elongation experiment, no significant differences in the root length and fresh weight were observed between the WT plants and the two transgenic lines (Figure 4D–F). In contrast, the transgenic seedlings maintained significantly longer roots and greater biomass than the WT plants under salt and drought stresses (Figure 4D–F).

### 2.4. Transgenic Arabidopsis Plants Display Better Chlorophyll Fluorescence Parameters Under Salt and Drought Stresses

**Figure 5:**
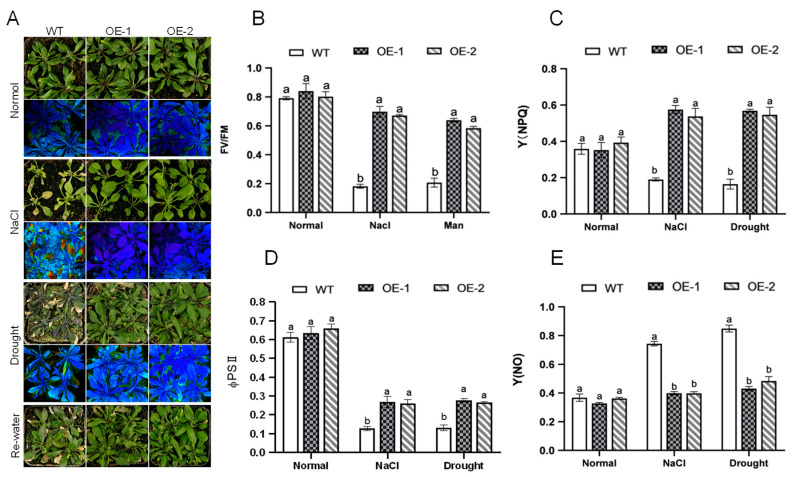
*AhNCED4* enhances tolerance to salt and drought stress in *Arabidopsis*. (**A**) The responses of *AhNCED4* transgenic and WT *Arabidopsis* plants were identified after two weeks of cultivation in pots under normal conditions, 300 mM NaCl stress, or drought stress followed by re-watering for three days. The maximal photochemical efficiency of photosystem II (PSII) was measured as (Fv/Fm). The false color code depicted at the bottom of the image ranges from 0 (red) to 1 (purple). (**B**–**E**) The chlorophyll fluorescence parameters, including Fv/Fm (**B**), Y(NPQ) (**C**), φPII quantum efficiency II (PSII) index (**D**), and Y(NO) (**E**) for WT and overexpressing *Arabidopsis* plants under normal, salt, and drought conditions. Data are presented as mean ± SE (*n* = 3). Different letters indicate significant differences between the same treatment groups.

To further investigate the function of *AhNCED4* in mature soil growth plants, 4-week-old seedlings of the transgenic and WT lines were subjected to salt and drought treatments. The tested plants all showed similar growth trends under normal conditions (Figure 5A). Under salt stress conditions, the wild-type (WT) plants exhibited nearly complete leaf yellowing, while the transgenic lines maintained over 50% of green foliage (Figure 5A). When subjected to drought stress (without irrigation), the WT plants showed extensive wilting of rosette leaves, contrasting with the transgenic *AhNCED4*-overexpressing lines, where senescence was primarily limited to mature basal leaves (Figure 5A). Notably, the WT plants failed to survive after a 2-day re-watering period, while the transgenic plantlets successfully recovered (Figure 5A). These phenotypic data indicated that *AhNCED4* overexpression enhances salt and drought tolerance in *Arabidopsis*.

To estimate photosynthetic responses under salt and drought stress, several chlorophyll fluorescence parameters, including Fv/Fm, ΦPSII, NPQ, and NO were compared between the WT and *AhNCED4* overexpressing *Arabidopsis* lines. We used the same transgenic lines grown in potting soil mixture to investigate abiotic stress tolerance. After treatment with normal conditions for two weeks, there were no significant differences in the chlorophyll fluorescence between the WT and transgenic lines. Following treatment with salt and drought stress, more leaves died in the WT plantlets than in the transgenic plantlets (Figure 5A). The survival rate, chlorophyll content, Fv/Fm, ΦPSII, and NPQ of the transgenic lines were significantly higher compared with the WT plants (Figure 5B–D). Conversely, the Y(NO) of overexpression lines was significantly lower than those of the WT plants (Figure 5E). These results demonstrate that *AhNCED4* overexpression preserves photosynthetic efficiency during abiotic stress through enhanced photoprotective capacity.

### 2.5. Overexpression of AhNCED4 Activated the ROS Scavenging System Under Salt and Drought Stress

**Figure 6:**
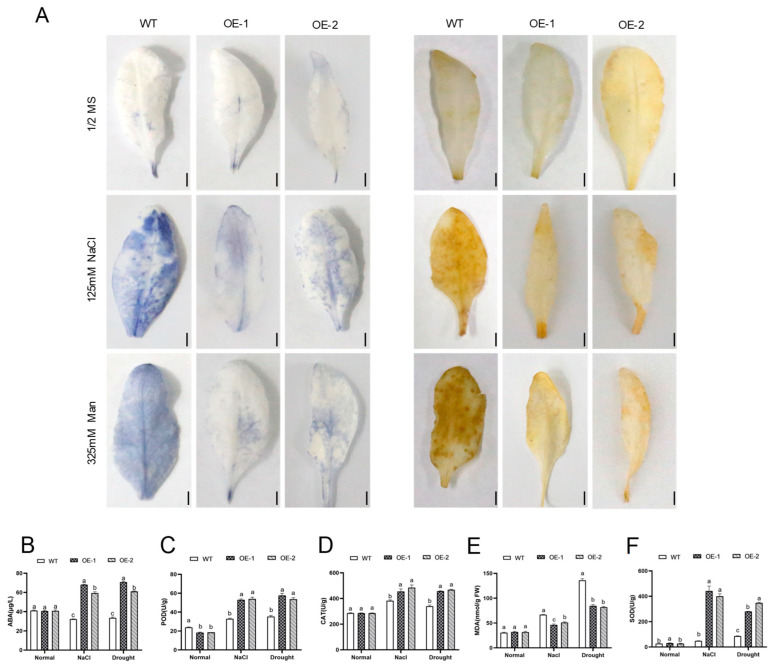
Detection of H_2_O_2_ and O^2−^ accumulation in transgenic and WT *Arabidopsis* plants under normal, salt, and drought stresses. (**A**) Leaves of *AhNCED4* transgenic and WT *Arabidopsis* plants were stained with NBT (**left**) and DAB (**right**) after three weeks of cultivation under normal conditions and NaCl and drought treatments. (**B**) The ABA hormone content. (**C**–**F**) The activity of enzyme SOD activity (**C**), POD activity (**D**), CAT (**E**), and MDA content (**F**) in WT and overexpression lines under normal conditions and NaCl and drought treatments. Data are presented as mean ± SE (*n* = 3). Different letters indicate significant differences between the same treatment groups.

Salt and drought stresses induce oxidative damage in plants through ROS accumulation. The ABA content was markedly higher in the transgenic lines than in the WT plantlets after stress treatment, and the content of the phytohormone did not significantly differ among the different lines without stress treatment (Figure 6B). In addition, the accumulations of two representative reactive oxygen species (ROS), H_2_O_2_ and O^2−^, were detected by DAB staining and NBT staining. Under normal conditions, the leaves of both the WT and *AhNCED4*-overexpressing lines exhibited only slight and comparable staining (Figure 6A), indicating that H_2_O_2_ and O^2−^ accumulated at low and similar levels in the leaves of both the WT and transgenic plants. However, after treatment with salt and drought stresses, significant staining differences became apparent between the WT and transgenic lines. The leaves of the WT plants developed more dense plaques compared to those of the overexpressing plants (Figure 6). These results indicate that the leaves of transgenic lines accumulated less H_2_O_2_ and O^2−^ than those of the WT plants.

In addition, to evaluate the physiological changes in the plants under salt and drought conditions, four physiological parameters, superoxide dismutase (SOD), peroxidase (POD), catalase (CAT), and malondialdehyde (MDA) levels, were measured. Under normal conditions, the overexpressing lines showed a similar accumulation of ROS compared to the WT plants (Figure 6C–F). However, after stress treatment, the transgenic lines showed significantly higher activities of SOD, POD, and CAT, as well as a lower MDA content compared with the WT plants (Figure 6C–F). Salt and drought stresses increased the H_2_O_2_ content in all types of plants but to a lesser extent in the overexpression lines. These results indicate that *AhNCED4*-overexpressing plants have an enhanced ROS-scavenging capacity.

### 2.6. AhNCED4 Activated Stress-Response-Related Genes

**Figure 7:**
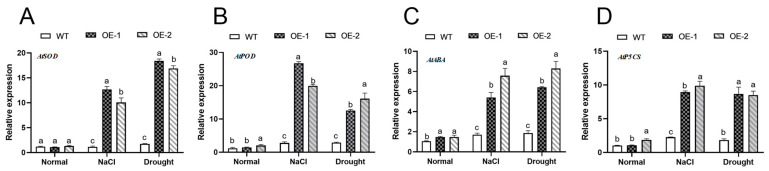
The relative expression levels of *AhNCED4* and abiotic-stress-responsive genes in the WT and transgenic *Arabidopsis* plants under normal salt and drought conditions. Expression levels of *AtSOD* (**A**), *AtPOD* (**B**), *AtABA* (**C**) and *AtP5CS* (**D**). Different letters indicate significant differences between the same treatment groups.

To further investigate how *AhNCED4* responds to drought and salt, the expression levels of certain ROS genes and stress-responsive genes were analyzed. Under normal conditions, no significant differences were observed in the expression levels of these genes between the transgenic and control lines (Figure 7A–D). Conversely, the expression of *AhNCED4* increased significantly following salt and drought treatment. Moreover, the expression of ROS genes (*AtSOD*, *AtPOD*), ABA, or proline-biosynthesis-associated genes (*AtABA1*, *AtP5CS*) was notably higher in the transgenic lines compared to the control lines (Figure 7A–D). These findings demonstrate that overexpression of *AhNCED4* enhanced the salt and drought stress tolerance of the plants by regulating the expression of stress-responsive genes related to ROS scavenging.

## 3. Discussion

The enzymatic activity of NCED family members mediates the conversion of cis-epoxycarotenoids to xanthoxin, a critical biochemical process that serves as a key regulatory node in the abscisic acid biosynthesis pathways of plants [31]. In the present study, a cDNA encoding *AhNCED4* was isolated from the salt-tolerant mutant M24. The expression of *AhNCED4* was strongly induced by NaCl, PEG6000, and ABA in peanut. *AhNCED4*-overexpressing transgenic plants demonstrated reduced malondialdehyde and peroxide levels, along with enhanced ROS scavenging capacity. Therefore, our results demonstrate that *AhNCED4* is crucial for peanut tolerance to salt and drought stresses.

In recent years, salinity and drought have increasingly constrained agricultural productivity and regional economies, disrupting plant physiological processes and limiting crop yields. As a key rate-limiting enzyme in abscisic acid biosynthesis, NCED proteins have been extensively studied for their regulatory roles in plant development and stress adaptation [34,35]. Despite this importance, the NCED gene family remains uncharacterized in peanut. In this study, a novel *AhNCED* gene was successfully identified and functionally characterized, demonstrating its critical involvement in salt and drought stress responses.

*NCED* serves as a critical regulator of abscisic acid (ABA) biosynthesis, directly activating downstream ABA signaling components [32,34]. In maize, the reduced ABA content in all the mutants is likely a result of downregulated *NCED4* expression, indicating a key role of *NCED4* in ABA biosynthesis and seed dormancy maintenance [36]. Previous studies have shown that overexpression of *OsNCED5* in *Oryza sativa* could increase ABA levels, enhanced tolerance to drought stress, and accelerated leaf senescence [37]. ABA-mediated stress responses involve synergistic interactions with jasmonic acid (JA) and salicylic acid (SA) pathways [38]. The overexpression of *BnNCED3* facilitates ABA accumulation, as well as the production of NO and ROS in transgenic *Arabidopsis* plants, consequently enhancing the plants’ tolerance to abiotic stress [28]. Consistent with these conserved mechanisms, in this study, overexpressing *AhNCED4* consistently increased tolerance to salt and drought stresses. The phenotypes observed in the transgenic lines were accompanied by significantly higher levels of ABA in *AhNCED4*-overexpressing transgenic plants. Therefore, the *AhNCED4* gene plays an important role in stress response.

In plants, salinity and drought induce oxidative stress in plants through reactive oxygen species (ROS) overaccumulation [39]. The excessive accumulation of ROS disrupts cellular homeostasis by impairing ion balance, damaging membrane integrity, inhibiting enzymatic activity, and degrading photosynthetic efficiency [5,40,41,42]. ROS-scavenging enzymes are major contributors to alleviating ROS damage, especially under various stresses [43,44,45,46]. Maintaining ROS equilibrium is critical for stress adaptation [47,48,49,50]. In our study, higher enzyme activities of SOD, POD, and CAT were detected in *Arabidopsis AhNCED4*-overexpressing lines when subjected to salt and drought treatment (Figure 6B–D). The transgenic lines exhibited lower levels of O_2_^−^ and MDA compared to the control plants, which indicates less membrane damage from excessive ROS (Figure 6E). ROS scavenging system-related enzymes were upregulated in overexpression lines under salt and drought treatment through qRT-PCR analysis. Furthermore, ABA modulates the activity of antioxidant enzymes (e.g., SOD, CAT) to regulate ROS levels. When ABA-induced ROS accumulation exceeds the scavenging capacity, it exacerbates thylakoid membrane damage, thereby further suppressing photosynthetic electron transport. Consequently, the enhanced capacity for ROS-scavenging likely contributes to the improved tolerance to salt and drought in the transgenic peanut and *Arabidopsis* lines.

Under stress treatment, ABA may influence chlorophyll metabolism through dual mechanisms: by promoting chlorophyllase activity while simultaneously suppressing the expression of chlorophyll-synthesis-related genes, ultimately leading to chlorophyll degradation. This process directly reduces the light-harvesting efficiency of photosystem II (PSII). Salt- and drought-stress-induced excessive reactive oxygen species (ROS) pose a threat to the photosynthetic apparatus, thereby impeding plant growth and development. Therefore, activation of ROS-scavenging mechanisms can effectively safeguard the photosynthetic machinery and the resistance of plants to salt and drought stress [44]. In our study, the chlorophyll content, Fv/Fm, ΦPSII, and NPQ of the transgenic lines were significantly higher compared with the WT plants under salt and drought stress. These findings demonstrate that *AhNCED4* overexpression strengthened the antioxidant defense system, consequently preserving photosystem integrity under salt and drought conditions.

## 4. Materials and Methods

### 4.1. Plant Materials

The salt-tolerant mutant M24 was developed through the Pingyangmycin mutagenesis of the “Huayu23” population followed by NaCl-directed screening. The wild-type (WT) *Arabidopsis thaliana* used in our study was ecotype Columbia (Col). The WT plant was cultivated under a light incubator (22 °C, 16 h light/8 h dark cycle) or on a half-strength MS medium for *AhNCED4* functional characterization through genetic transformation.

### 4.2. AhNCED4 Expression Analysis in Peanut

The total RNA was extracted from 30-day-old leaves and roots of huayu20 using TRIzol reagent (Invitrogen, Carlsbad, CA, USA) followed by purification using a Qiagen RNeasy Kit (Qiagen, Hilden, Nordrhein-Westfalen, Germany). First-strand cDNA synthesis was conducted using a Superscript™ II 1st Strand cDNA Synthesis Kit (TaKaRa, Beijing, China). To analyze the expression of *AhNCED4* under different stress conditions, the 3-week-old seedlings of huayu20 were treated with 300 mM NaCl, 30% PEG6000, and 100 μM ABA at different times (0, 1, 3, 6, 12, and 24 h). The expression levels were analyzed using a CFX96 Real-Time System (Bio-Rad, Hercules, CA, USA) with *AhActin* as the internal reference control. The variation in expression was estimated from three biological replicates, analyzing the relative gene expression data by the 2^−ΔΔCt^ method.

### 4.3. Cloning and Sequences Analysis of AhNCED4

According to the sequence alignment, *AhNCED4*-specific primers AhNCED4-F/R were designed from the *AhNCED4* coding sequence. Similarities in protein sequences were analyzed using the BLAST program on the NCBI website, according to the GenBank database (https://www.ncbi.nlm.nih.gov//Blast.cgi, accessed on 15 March 2023). Multiple sequence alignment of NCED4 from different plants was conducted with the DNAMAN 7 software (Table S2). For the phylogenetic tree construction, MEGA11 was used with the neighbor-joining method [51].

### 4.4. Vector Construction and Arabidopsis Transformation

The full-length coding sequence (CDS) of *AhNCED4* was amplified from salt-tolerant mutant M24 and cloned into the SuperpCAMBIA1300 vector via the *Kpn*Ⅰ/*BamH*Ⅰ restriction sites to generate the SuperpCAMBIA1300-*AhNCED4* overexpression construct. This recombinant vector was introduced into wide-type *Arabidopsis* using the *Agrobacterium*-mediated floral dip [52]. Transgenic *Arabidopsis* seeds were planted on 1/2 Murashige and Skoog (MS) media containing 25 mg/L hygromycin, with two T_3_ homozygous lines (OE-1 and OE-2) selected for phenotypic analysis.

### 4.5. Drought and Salt Tolerance Analysis of Arabidopsis Transgenic Lines

To perform the germination assay, WT and transgenic *Arabidopsis* seeds (these seeds were cultivated under a light incubator 22 °C 16 h light/8 h dark cycle) and were planted on 1/2 MS medium containing either 125 mM NaCl or 325 mM mannitol, respectively. A seed was regarded as germinated when the radicle tip emerged from the seed coat, and each culture dish contained 50 seeds. The germination rate and green rate of cotyledons were counted within 7 days. Seven-day-old *Arabidopsis* seedlings of transgenic and WT lines were transferred to 1/2 MS medium supplemented with 100 mM NaCl or 350 mM mannitol, and the root length and fresh weight were measured after 10 days. Transgenic *Arabidopsis* and WT plantlets were grown in soil (a mixed substrate composed of substrate soil–vermiculite–perlite at a volume ratio of 2:2:1) for further growth for three weeks. The experiments were conducted in plastic pots with cubic dimensions of 7 cm (length) × 7 cm (width) × 7 cm (height). Uniform seedlings were then watered with 300 mM NaCl solution (200 mL of NaCl solution was applied for salinization) every two days for three weeks or were not watered for three weeks followed by re-watering for two days, respectively.

### 4.6. Histochemical Detection of ROS

ROS accumulation in *Arabidopsis* leaves under normal/stress conditions was visualized through 3,3′-diaminobenzidine (DAB) and nitroblue tetrazolium chloride (NBT) staining following established protocols [53]. Fresh leaves were collected from salt-/drought-treated WT and transgenic *Arabidopsis* plants. The activity of superoxide dismutase (SOD), peroxidase (POD), and catalase (CAT) was measured according to the manufacturer’s instruction (SOD: AKAO001M, boxbio, Beijing, China; POD: AKAO005M, boxbio, Beijing, China; CAT: AKAO003-2M; boxbio, Beijing, China), and the content of MDA was measured using the relevant chemical kits (Suzhou Grace Biotechnology Co, Suzhou, China). The detailed experimental procedures were conducted as follows: The nitrotetrazolium (NBT) photochemical reduction method: SOD inhibited the photochemical reduction reaction of NBT under light by catalyzing the disproportionation of superoxide anion radicals (O^2−^) into H_2_O_2_ and O^2−^. The NBT reduction product had a characteristic absorption peak at 560 nm, and the absorbance was negatively correlated with SOD activity. The guaiacylphenol method: In the presence of H_2_O_2_, POD catalyzed the oxidation of guaiacol to generate red–brown products. The absorbance change was measured at 470 nm, and the increase in absorbance was positively correlated with the enzyme activity. CAT decomposed H_2_O_2_ to generate H_2_O and O_2_. The enzyme activity was calculated by monitoring the absorbance attenuation rate (ΔA/min) of H_2_O_2_ at 240 nm. The thiobarbituric acid (TBA) method: MDA was heated with TBA under acidic conditions (95 °C, 30 min) to form a reddish-brown product (Mikagawa). The absorbance was measured at 532 nm, and the non-specific absorption was measured at 600 nm, and the background was subtracted simultaneously. The MDA content was calculated based on the molar extinction coefficient.

### 4.7. Determination of Chlorophyll Fluorescence in Arabidopsis

Chlorophyll fluorescence parameters were measured afterwards using the IMAG-K7 chlorophyll fluorescence imaging system (WALZ, Bavaria, Nuremberg, Germany). The maximal photochemical efficiency of photosystem II (PSII) (Fv/Fm), the quantum efficiency of PSII photochemistry (ΦPSII), the non-photochemical quenching (NPQ), and the photodamage index (NO) were calculated according to the formulas described by Kramer et al. (2004) [54]. The parameter calculation formula is as follows: Fv/FM = (FM − Fo)/FM5; ΦPSII = (FM’ − Fs)/FM’ = ΔF/FM’; NPQ = (FM − FM’)/FM’ = FM/FM’ − 1; qP = (FM − FS)/(FM’ − Fo’).

### 4.8. Expression Analysis of Stress-Related Genes

The leaves of *AhNCED4* transgenic *Arabidopsis* plants were collected to detect gene expression under normal, salt, and drought conditions. qRT-PCR quantification assessed expression patterns of ROS-scavenging genes and stress-response genes. The *AhActin* gene was used as an internal control.

### 4.9. Primers

The primers used in this study are listed in Table S3.

### 4.10. Statistical Analysis

All experiments were repeated three times, and data are presented as means ± SE. Two-tailed Student’s *t*-tests were performed using SPSS version 17 (SPSS Inc., Chicago, IL, USA). Statistical significance was set at *p* < 0.05 and *p* < 0.01.

## 5. Conclusions

Peanuts are an important economic and oil crop, providing oil and protein for human nutrition. In this study, the *AhNCED4* gene was shown to play an important role in salt and drought tolerance. Transgenic plants of *AhNCED4* exhibited lower malondialdehyde and peroxide contents as well as a higher ability to remove ROS. These findings establish that *AhNCED4* confers stress tolerance through coordinated upregulation of antioxidant enzymes and stress-responsive genes. This result may unveil that a novel gene, *AhNCED4*, has the potential to improve the abiotic stress tolerance of peanut, although the molecular mechanisms underlying salt and drought tolerance warrant further investigation. Therefore, our results demonstrate that *AhNCED4* is crucial for peanut tolerance to these stresses. Moreover, the potential of *AhNCED4* in genetically improving crops to better withstand the challenges posed by climate change cannot be overstated.

## Data Availability

Data is contained within the article.

## Supplementary Materials

The following supporting information can be downloaded at: https://www.mdpi.com/article/10.3390/plants14121741/s1, Table S1: The full-length *AhNCED4* gene sequence with its coding and protein sequences in peanut; Table S2: Protein sequences of homologous NCED4 from eight different plant species; Table S3: Primes used in this study.
